# Ventricular tachycardia in repaired congenital heart disease

**DOI:** 10.1007/s00399-016-0428-4

**Published:** 2016-05-19

**Authors:** Katja Zeppenfeld

**Affiliations:** Department of Cardiology, C5-P, Leiden University Medical Centre, PO Box 9600, 2300 RC Leiden, The Netherlands

**Keywords:** Ventricular tachycardia, Repaired congenital heart disease, Tetralogy of Fallot, Catheter mapping, Radiofrequency catheter ablation, Ventrikuläre Tachykardie, Korrigierte angeborene Herzerkrankungen, Fallot-Tetralogie, Katheter-Mapping, Radiofrequenzkatheterablation

## Abstract

Ventricular arrhythmias are an important cause of late morbidity and sudden cardiac death in the growing population of adults with repaired congenital heart disease. Risk stratification remains challenging because of the heterogeneity of the malformations and the surgical approaches. Therapeutic interventions depend on the type of ventricular arrhythmia, which can be polymorphic ventricular tachycardia (VT) or ventricular fibrillation in patients without ventricular scars, but also potentially fatal monomorphic reentrant VTs, typical for patients with ventricular scars or obstacles. Advances in surgical techniques have improved survival and have important implications for the arrhythmia substrates and prognosis. Over the past few decades, progress has been made to determine the anatomical basis for monomorphic VT in patients with ventricular surgical scars and patch material. These substrates can be currently identified and targeted during sinus rhythm by radiofrequency catheter or surgical ablation without the need for VT induction. The review provides an update on the evolving surgical approaches, the changing VA substrates, and the potential consequences for individualized risk assessment and tailored treatment.

## Zusammenfassung

In der an Zahl zunehmenden Gruppe Erwachsener mit einer korrigierten angeborenen Herzerkrankung sind Kammerrhythmusstörungen eine wichtige Ursache für späte Morbidität und plötzlichen Herztod. Eine Risikostratifizierung gestaltet sich i. Allg. schwierig aufgrund der Heterogenität der Fehlbildungen und der chirurgischen Eingriffe. Therapeutische Interventionen sind abhängig von der Art der Kammerrhythmusstörung. Polymorphe ventrikuläre Tachykardien (VT) und Kammerflimmern können bei Patienten ohne chirurgische Narben oder eingebrachtes Fremdmaterial im Ventrikel auftreten, während potenziell letale monomorphe Reentrytachykardien typisch für Patienten nach chirurgischen Interventionen an den Hauptkammern sind. Technische Weiterentwicklung führte nicht nur zur Verbesserung der Überlebenschance, sondern auch zu einem wichtigen Einfluss auf die Entwicklung des spezifischen arrhythmogenen Substrats und der Prognose. In den letzten Jahrzehnten wurden große Fortschritte darin erzielt, die anatomischen Grundlagen für monomorphe Reentrytachykardien bei Patienten mit korrigierten angeborenen Herzerkrankungen zu identifizieren. Dieses Substrat für VT kann während eines stabilen Sinusrhythmus rekonstruiert und die identifizierten Gebiete mittels Radiofrequenzkatheterablation oder während eines operativen Eingriffs behandelt werden, ohne eine VT zu induzieren. Im vorliegenden Übersichtsartikel werden die Veränderungen und Konsequenzen chirurgischer Interventionen, die wichtigsten neuen Erkenntnisse über Substrate von Kammertachykardien und die sich daraus ergebenden Möglichkeiten der individualisierten Risikostratifizierung und Therapieentscheidung zusammengefasst.

The population of adults with repaired congenital heart disease (rCHD) is growing [21]. Surgical approaches and the timing of intervention have changed over the past few decades with better survival in infancy and a trend toward death at older age [15]. Improved survival exposes more patients to the risk of late ventricular arrhythmias (VA) contributing to morbidity and sudden cardiac death (SCD). Knowledge of the individual malformation, the type of repair, and the related VA is a prerequisite for risk stratification and therapeutic interventions.

## Type and mechanism of VA

Although sudden death may occasionally be due to cerebral, vascular, or thrombo-embolic events, the majority is assumed to be arrhythmic SCD [19]. Life-threatening arrhythmias rarely occur during childhood, and a time-dependent incremental risk for VA and SCD has been observed particularly in patients with repaired tetralogy of Fallot (rTOF) [6].

Although systemic ventricular dysfunction is a dominant predictor for SCD in unselected populations of adults with CHD, two thirds of those who die suddenly or experience life-threatening VTs have a preserved cardiac function prior to the event [4, 5, 19, 24]. These data suggest that different VA mechanisms may be operative.

VAs include monomorphic ventricular tachycardia (MVT), polymorphic VT, and ventricular fibrillation. It is crucial for risk stratification and treatment to understand and identify the underlying substrate for these different VAs. VAs can occur in the absence of any surgical scar or patch material, typical for patients with left heart obstruction or atrial switch operation for simple dextro-transposition of the great arteries (d-TGA).

However, MVT in these patients is uncommon. The estimated incidence rate for MVT in an unselected group of patients after atrial switch for d‑TGA was 0.5 % per year [24]. The majority of documented VAs in d‑TGA patients who have received an implantable cardioverter defibrillator (ICD) were polymorphic VT or ventricular fibrillation [13].

The underlying arrhythmia mechanisms are likely to be similar to those observed in other cardiac diseases with pathologic hypertrophy, fibrosis, progressive right or left ventricular dilatation, and eventually heart failure. Hypertrophy can be due to chronic pressure overload in left heart obstructions. Progressive ventricular dysfunction usually occurs if the right ventricle (RV) serves as the systemic ventricle after atrial switch operation for d‑TGA or in congenitally corrected transposition of the great arteries (ccTGA). The therapeutic options comprise treatment of residual lesions, optimal heart failure management to reduce further adverse remodeling, and ICD implantation. Specific anti-arrhythmic drugs or catheter or surgical interventions are not available.

By contrast, in the presence of surgical scar and patch material, MVTs are the most common type of observed VAs. MVT after repair of TOF can serve as a paradigm of these postoperative arrhythmias [11, 20, 26].

Indeed, more than 80 % of all treated VAs in rTOF patients who have received ICDs for primary and secondary prevention were monomorphic and fast VTs with a median heart rate of 212 bpm [14]. These fast VTs can be fatal, even in the presence of a preserved cardiac function. MVTs have also been described after ventricular septal defect (VSD) closure and repair of complex d‑TGA. The latter is associated with VSD in approximately 40 % of cases, or less frequently with pulmonary outflow tract obstruction, and may provide a similar substrate for macro-reentrant VTs than those observed in rTOF.

**Fig. 1:**
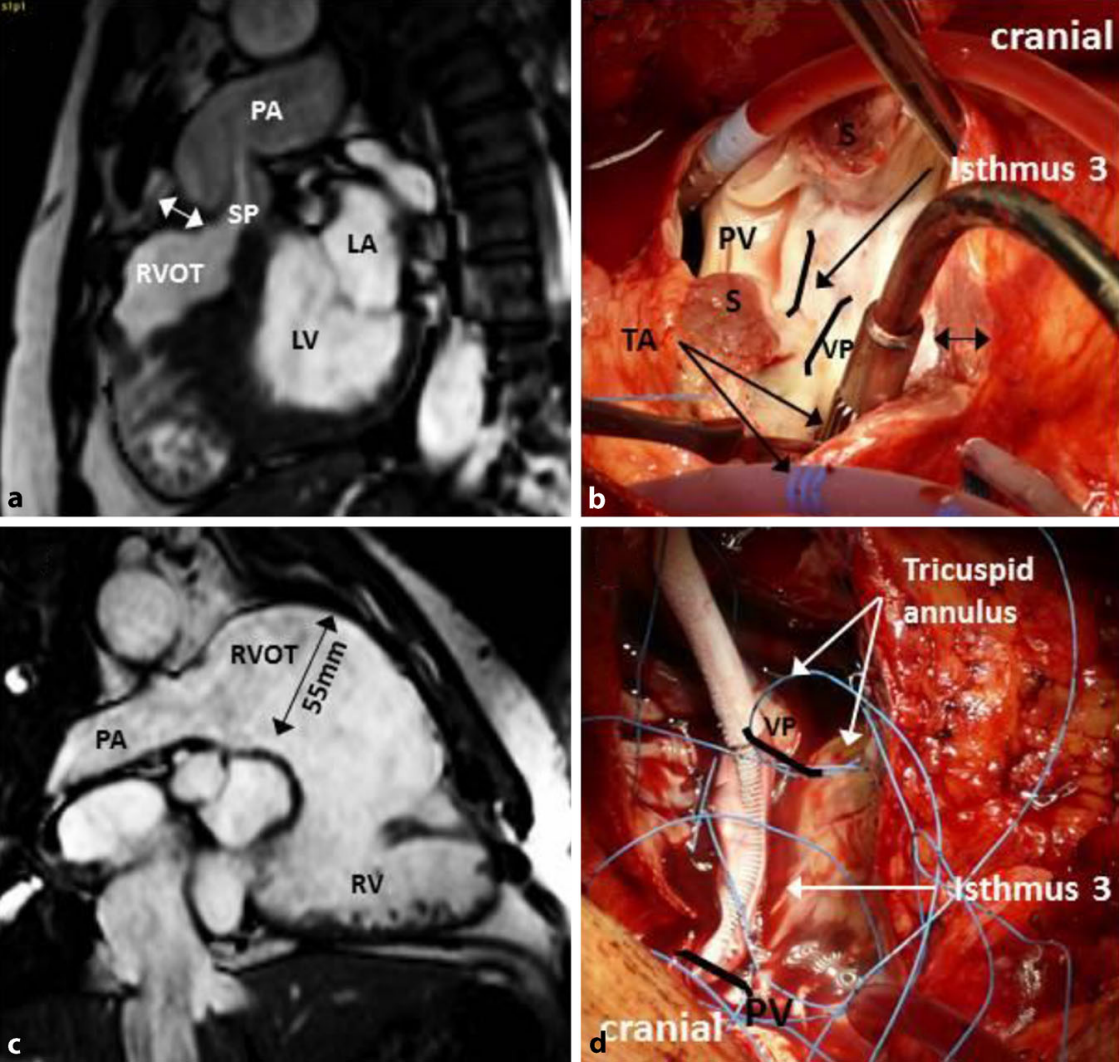
Cardiac magnetic resonance imaging (MRI; **a**) and intraoperative findings (**b**) of a patient with repaired tetralogy of Fallot (rTOF) and residual subpulmonary stenosis without ventricular tachycardia (VT). Isthmus 3 bordered by the ventricular septal defect (VSD) patch and the PV consisting of fibrous tissue precluding its participation in VT. **c** Cardiac MRI of a patient with rTOF and severe PV regurgitation with RVOT dilatation and VT related to a slow-conducting anatomical isthmus 3. Intraoperative cryoablation of isthmus 3 connecting the PV and the VSD patch (**d**). *PA* pulmonary artery, *SP* subpulmonary stenosis, *RVOT* right ventricular outflow tract, *LA* left atrium, *LV* left ventricle, *PV* pulmonary valve, *TA* tricuspid annulus, *VP* VSD patch

After initial repair of TOF, RV hypertrophy may develop as a result of residual RVOT obstructions. Progressive RV dilatation and dysfunction can be the consequence of chronic pulmonary regurgitation, which is more likely if initial repair is performed through a right ventriculotomy combined with the use of large transannular patches (Fig. 1). These patients may be at risk for both polymorphic VT/ventricular fibrillation and macro-reentrant VT.

## Anatomical substrate for macro-reentrant VT

**Fig. 2:**
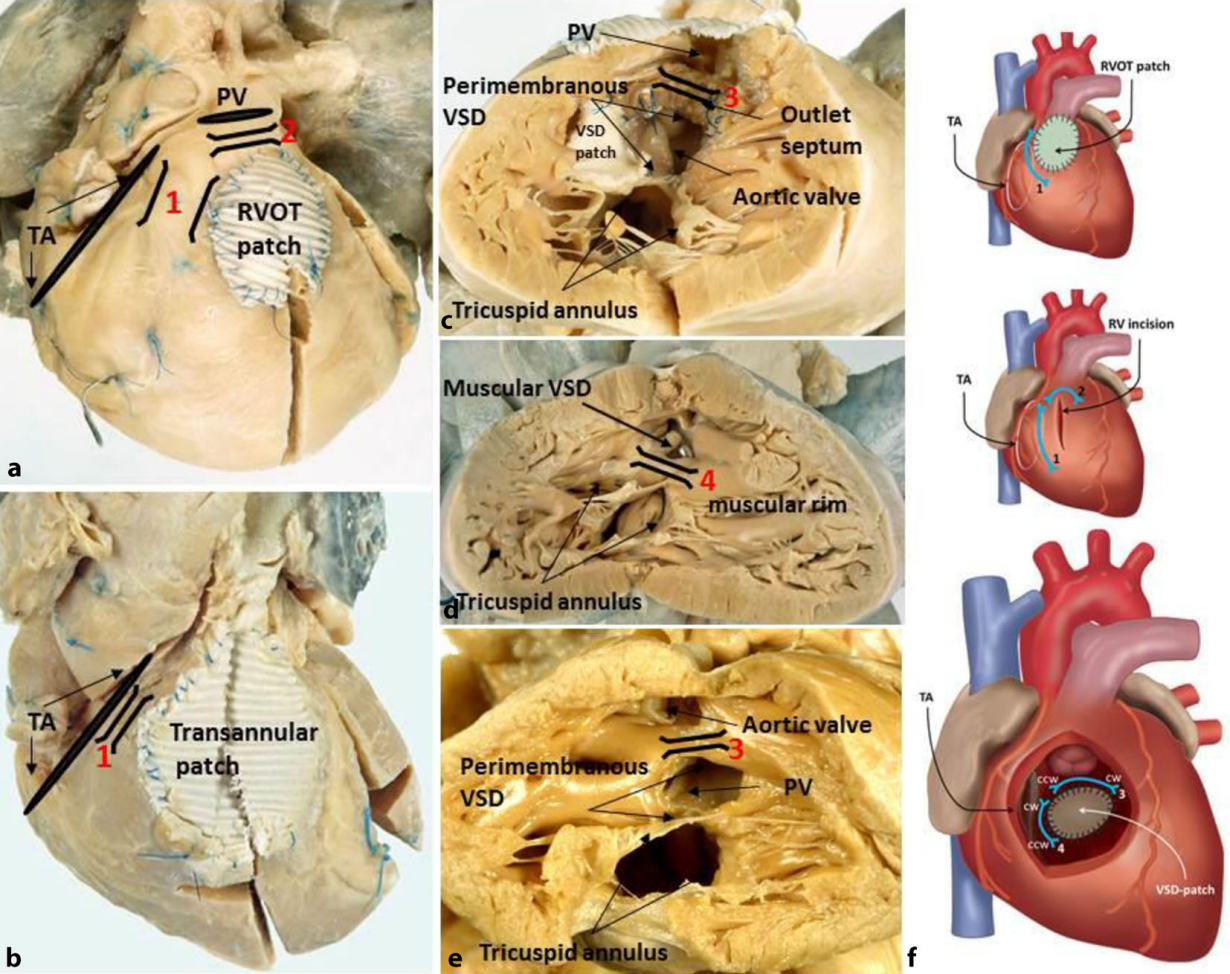
Postmortem specimens of repaired tetralogy of Fallot (rTOF; **a–c**), unrepaired TOF (**d**), and unrepaired dextro-transposition of the great arteries (**e**). Anterior views (**a,** **b**), right ventricular views (**c–e**). Anatomical isthmuses (AIs) *1–4* are indicated. Unexcitable borders are (potential) patches (ventricular septal defect patch is folded back in **c**), PV and TA. Schematic of AIs 1–4 in rTOF (**f**). *PV* pulmonary valve, *TA* tricuspid annulus, *RVOT* right ventricular outflow tract, *VSD* ventricular septal defect

Areas of dense fibrosis after surgical incisions, patch material, and the valve annuli form regions of conduction block that can define reentry circuit borders and create intervening anatomical isthmuses (AIs) consisting of conducting myocardium [11, 26]. After repair of TOF, four AIs related to VT have been described, whose presence is dependent on the variation of the malformation and the type of repair (Fig. 2; [26]). Isthmus 1 is bordered by the tricuspid annulus and the transversal or longitudinal surgical RV incision or the anterior RVOT transannular patch; isthmus 2 by the pulmonary annulus and the RV incision or an RVOT patch sparing the pulmonary valve annulus; and isthmus 3 is located between the pulmonary annulus and the VSD patch. Finally, isthmus 4 is bordered by the VSD patch and the tricuspid annulus in the about 20 % of TOF patients with muscular VSDs. The majority, however, have a perimembranous VSD, excluding isthmus 4. Accordingly, in two postmortem series of TOF, isthmus 3 and 1 were present in almost all specimens, whereas isthmus 2 and 4 were less frequently observed [22, 26]. Of interest, isthmus 3 may also be present in complex TGA associated with VSDs (Fig. 2e) and in patients with isolated VSD. Importantly, not all AIs are related to VT [11]. Macro-reentrant VTs are facilitated by slow conduction often observed in areas with intervening fibrosis. Progressive interstitial fibrosis may be due to longstanding cyanosis in those who undergo repair at an older age and/or due to pressure or volume overload in those with residual or new lesions, but may also be the consequence of aging [3]. Of interest, remodeling over time may also be dependent on the initial characteristics of the AI. In a postmortem series of rTOF patients who survived surgery, isthmus 3 was significantly narrower and thinner with more interstitial and replacement fibrosis than isthmus 1, perhaps predisposing isthmus 3 to further arrhythmogenic remodeling [22]. Indeed, in rTOF patients who underwent VT ablation, isthmus 3 was more frequently related to VT than the other AIs [11].

The coincidence of unexcitable anatomical boundaries and pathological remodeling over time may explain the high incidence of late macro-reentrant VT in particular in patients with rTOF.

## Risk assessment in contemporary patients with rCHD

Most data on risk stratification are derived from patients with rTOF. Retrospective, observational studies have identified several factors associated with VA and SCD (Tab. 1). Currently, primary prevention ICD implantation is considered reasonable in selected adults with TOF and multiple risk factors for SCD, such as left ventricular (LV) systolic or diastolic dysfunction, nonsustained VT, a QRS width of ≥180 ms, extensive RV scarring, or inducible sustained VT at electrophysiological study [1, 17].

**Tab. 1:** Reported risk factors for VT and/or SCD in rTOF

Older age at repair
Transannular patch
RV parameter (moderate to severe dysfunction, severe dilatation, LGE, akinetic region length, mass/volume ratio ≥0.3 g/ml)
Moderate to severe PVR
LV parameter (moderate to severe dysfunction, longitudinal strain, end-diastolic pressure)
History of atrial arrhythmias
(Pre)Syncope
QRS duration (≥180 ms), QRS duration increase per year
Nonsustained VT
Inducible for MVT/PVT at PES

*RV* right ventricle, *LGE* late gadolinium enhancement, *PVR* pulmonary valve regurgitation, *LV* left ventricle, *VT* ventricular tachycardia, *MVT* monomorphic ventricular tachycardia, *PVT* polymorphic ventricular tachycardia, *PES* programmed electrical stimulation, *rTOF* repaired tetralogy of Fallot, *SCD* sudden cardiac death

Risk stratification in patients after the atrial switch operation for d‑TGA is even more challenging. Symptoms of arrhythmias and heart failure and documented atrial flutter or fibrillation have been identified as predictors of SCD. In addition, sustained VT and SCD are more likely in patients with additional anatomical lesions, impaired RV function, NYHA class III or greater, and a QRS duration of ≥140 ms [10]. Primary prevention ICD implantation may therefore be reasonable in patients with a systemic RV ejection fraction of <35 %, particularly in the presence of additional risk factors [1, 17]. However, most of the aforementioned risk factors have only limited predictive value for an individual patient and may no longer apply to the majority of contemporary patients with rCHD.

The currently applied arterial switch operation for d‑TGA disconnects the aorta and pulmonary trunk from their arterial roots and connects them to the correct ventricle. The LV serves as systemic ventricle that may prevent the occurrence of late VA.

Repair of TOF has changed from a late transventricular repair after palliative shunt operation to a combined transatrial–transpulmonary approach, now usually performed early in life. The latter approach may prevent severe RV hypertrophy, RV and LV dysfunction, and increased intraventricular pressure and fibrosis, all factors associated with late VA and SCD. In addition, AIs 1 and 2 may also be prevented by the modern approach, whereas isthmus 3 and occasionally isthmus 4 cannot be avoided. In those patients, a positive programmed electrical stimulation (PES), defined as inducibility of a sustained MVT, may remain an important tool to prove the presence of a substrate for reentrant VT. Inducibility strongly depends on the applied induction protocol often requiring three extra stimuli and occasionally isoproterenol [16]. Of interest, inducibility of the clinical VT could be achieved in all patients with rCHD referred for VT ablation if stimulation was performed from a site close to the infundibular septum [11].

A wide QRS (≥180 ms) and an increase in QRS duration have consistently been reported as important risk factors for both MVT and SCD [6]. QRS prolongation has been associated with RV dilatation and arrhythmogenesis explained by a mechanoelectrical interaction [7]. However, a prolonged QRS duration may be explained not only by global conduction delay in a dilated RV. It can also be due to morphological and functional changes restricted to the RVOT and may reflect conduction delay within AIs [25]. This is further supported by the finding that surgical pulmonary valve replacement (PVR) has been associated with a reduction in RV volume but not with a consistent reduction in QRS duration after surgery [2].

In addition, replacing the valve did not eliminate the risk for MVTs, despite postoperative improvement in RV volumes and function [9]. An empirical surgical cryoablation lesion at the time of PVR, connecting the boundaries of the AIs, has shown promising results in treating and preventing MVT and has been suggested as alternative to mapping-guided intraoperative ablation [23].

## Treatment for VT

**Fig. 3:**
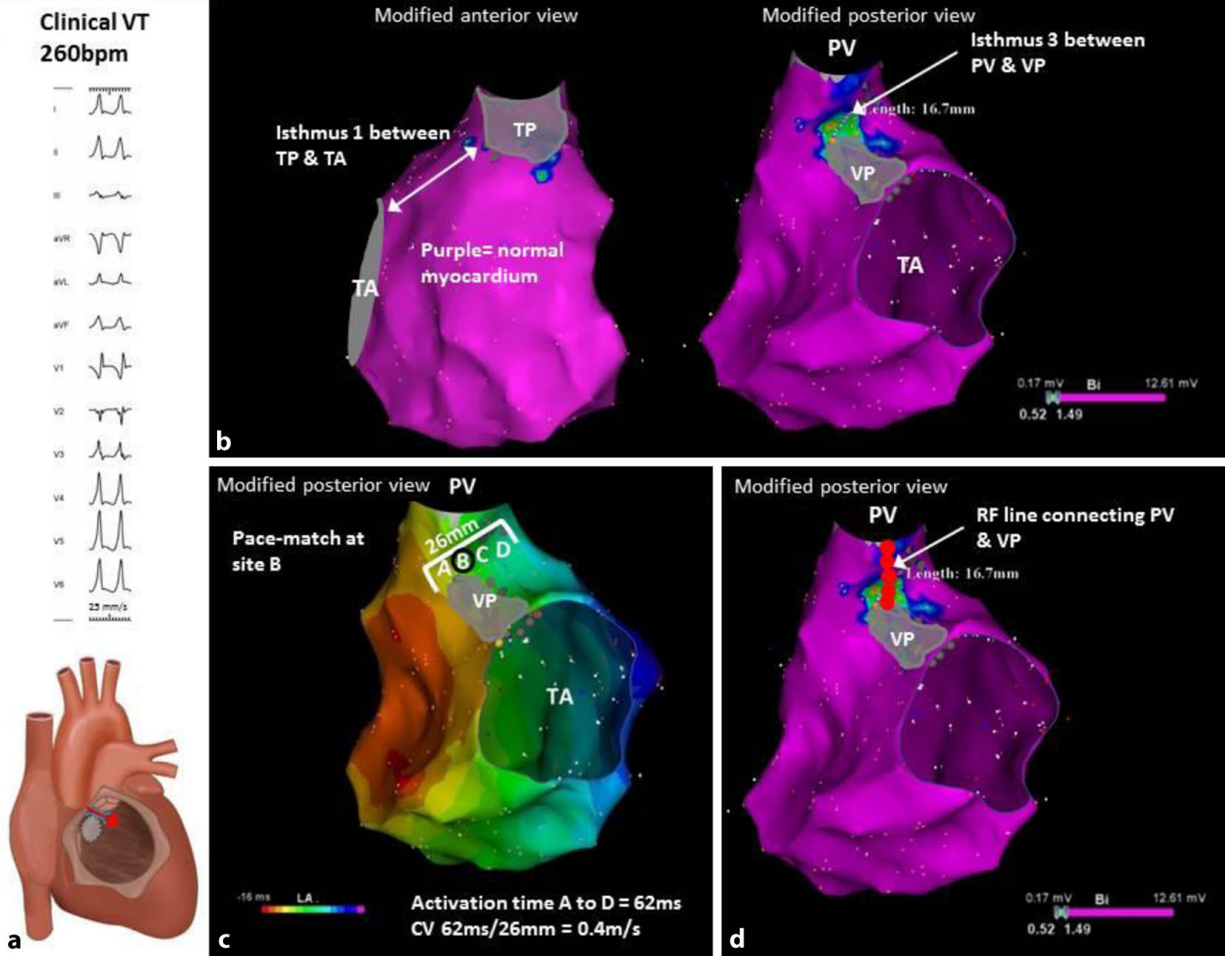
**a** Clinical, fast ventricular tachycardia (VT). Please note QR pattern in V_1_, consistent with clockwise propagation through anatomical isthmus (AI) 3 bordered by the pulmonary valve (*PV*) and the ventricular septal defect patch (*VP*). **b** Voltage map with a wide AI I (normal voltage) bordered by a small transannular patch (TP) and tricuspid annulus (*TA*) and a narrow AI 3 (width, 16.7 mm). **c** Activation map during sinus rhythm. Isthmus 3 was the only slow-conducting AI (conduction velocity = 0.4 m/s) and related to VT, as demonstrated by pace-mapping (pace-match of VT was obtained at site B; not shown). **d** Connecting the PV and VP with a linear RF lesion resulted in blocking of AI 3 preventing VT re-induction and VT recurrence

Patients with rCHD and VA may be SCD survivors or present with often highly symptomatic, fast VTs (Fig. 3). According to current guidelines, an ICD should be implanted in SCD survivors after exclusion of a reversible etiology. Of importance, 70 % of the patients with rTOF who have received an ICD for primary or secondary prevention required ICD shocks to terminate the first arrhythmia [14]. Even if ATP was programmed and delivered appropriately, it failed in 81 % of patients with rCHD [18]. Therefore, additional therapeutic interventions are needed.

There are no specific data available on the efficacy of antiarrhythmic drugs in rCHD. In general, the available drugs to prevent recurrence of reentrant VT in structural heart disease have limited efficacy. Sotalol or Amiodarone may be effective in reducing appropriate ICD therapy but their use is often hampered by serious side effects leading to drug discontinuation. The feasibility of radiofrequency catheter ablation (RFCA) in patients with rCHD was reported 20 years ago, the majority performed in TOF patients, followed by patients with VSD closure and atrial switch for complex d‑TGA [8]. Initially only slow and hemodynamically tolerated VT could be targeted, approachable by conventional mapping techniques, such as activation and entrainment mapping during ongoing VT.

Over the last decade, progress has been made in identifying the anatomical substrate for fast VTs, which is currently approachable by substrate-based ablation techniques [11, 20, 26]. RFCA is perhaps the most important treatment option, which should be considered as adjunct to ICD therapy. Although an ICD is often indicated in patients with spontaneous sustained VT, catheter ablation or surgery may even offer a reasonable alternative to ICD therapy in carefully selected patients [11, 23].

Up to 97 % of all spontaneous and induced monomorphic VTs in the contemporary population of patients with rCHD referred for RFCA or studied for risk stratification are macro-reentrant VTs with a critical reentry circuit isthmus located within anatomically defined isthmuses [11].

These AIs can be reconstructed with point-by-point electroanatomical voltage mapping during stable sinus rhythm using three-dimensional electroanatomic mapping systems (Fig. 3). Peak-to-peak bipolar electrogram amplitudes can be displayed color-coded as a voltage map and projected on a three-dimensional shell of the RV. In addition, local activation times derived from bipolar and unipolar electrograms can be displayed as an activation map. Electrograms greater than 1.5 mV are considered normal voltage. At sites with amplitudes less than 0.5 mV, high-output pacing (10 mA, 2 ms) can be performed to identify unexcitable tissue. Noncapture sites, consistent with patch material or surgical scars, serve as boundaries of AIs [11, 26].

The critical reentry circuit isthmus of each induced VT can be occasionally determined by activation and entrainment mapping for tolerated VT. However, considering the often hemodynamically unstable VTs, pace-mapping within AIs is often applied to determine whether the critical isthmus is likely located within an anatomically defined isthmus. The VT-related AI can be transected by connecting the adjoining anatomic boundaries by a linear RF lesion [26].

An alternative approach for mapping of poorly tolerated VTs has applied noncontact mapping [20]. The system, consisting of a multielectrode balloon array, allows for the simultaneous acquisition of virtual unipolar electrograms and recording of the activation sequence requiring only a single VT beat. The VT circuit and the critical VT isthmus can be displayed on the anatomic shell and the corresponding AI can be targeted by RFCA.

Demonstration of conduction block after transection of an AI is an accepted and clearly defined procedural endpoint. Accordingly, noninducibility of any VT *and* transection of the critical AI was defined as complete procedural success and achieved in 25 of 34 adults with VT after CHD repair. None of these patients had recurrence of a monomorphic VT during 46 ± 29 months of follow-up [11]. These data strongly support the concept that specific “arrhythmogenic” AIs are the substrate for macro-reentrant VT in rCHD that can be effectively treated by catheter ablation. Confirmed conduction block of the arrhythmogenic AI should be attempted and, if successful, may be considered curative in patients with preserved cardiac function and no competing heart failure-related VA mechanism.

## Arrhythmogenic anatomical isthmuses

The 12-lead morphology of the clinically documented or induced VT may already provide an indication of the AI being involved (Fig. 3). However, specific electroanatomical characteristics, including isthmus width and conduction velocity through an AI, may allow for direct identification of the “arrhythmogenic” AI that needs to be targeted by ablation. This information is available after the three-dimensional reconstruction of voltage and activation maps during stable sinus rhythm. In a cohort of 74 TOF patients, only electroanatomical narrow and slow-conducting AIs (calculated conduction velocity <0.5 m/s) were the substrate for all 37 documented and induced MVTs in 24 patients with preserved cardiac function (unpublished data). The inability to transect an AI may be due to the hypertrophied myocardium preventing transmural lesions. Perhaps more important, patch material or a pulmonary homograft implanted for late pulmonary valve regurgitation may cover parts of the arrhythmogenic isthmus 3. Occasionally, a left-sided approach from the aortic root or LV outflow tract can be successful for transecting this isthmus [12]. In patients who need to undergo re-operation, preoperative PES and electroanatomical mapping followed by preventive intraoperative ablation of potentially arrhythmogenic AIs should therefore be considered.

## Conclusion

Risk stratification and treatment of complex VAs in patients with rCHD remain challenging and require a multidisciplinary approach. Progress has been made in understanding and delineating the substrate for MVT. The strong link between slow-conducting AIs and the often poorly tolerated VTs allows for RFCA without VT inducibility and (preventive) surgical ablation in those who need surgical re-interventions. Substrate identification may overcome the problem of lacking clinical arrhythmia predictors and could facilitate personalized risk stratification and tailored treatment in the contemporary population of patients with rCHD.
